# From topography to connectome: Towards an integrated understanding of the resting brain

**DOI:** 10.64898/2026.06.06.730493

**Published:** 2026-06-08

**Authors:** Samuel Naranjo Rincon, Fyzeen Ahmad, Ty Easley, Shirin Shoushtari, Tristan Glatard, Gregory Kiar, Hailey Modi, Simon Dahan, Emma Robinson, Ulugbek Kamilov, Janine Bijsterbosch

## Abstract

As the field expands from early research into the human connectome, there has been a fast expansion in the number of analytical approaches to study resting state functional MRI (rsfMRI) data. With increasing focus on individual differences, topographical brain maps of spatial organization have emerged in addition to traditional functional connectomes. Here, we developed a deep-learning model to embed maps of network topography and faithfully translate to individualized connectomes. Results confirmed the validity of the surface vision transformer based on reconstruction accuracy (0.73±0.09) and accurate topography-to-connectome translation (0.43±0.08). Importantly, translated connectomes retained identifiability and brain-cognition associations. These findings establish a direct mapping from spatial topography to connectomes that can be used to integrate scientific insights across rsfMRI sub-fields. This is an important step towards broadening our conceptualization of the connectome and supporting broader integration of findings to inform a complete understanding of the human connectome.

## Full Text Availability

The license terms selected by the author(s) for this preprint version do not permit archiving in PMC. The full text is available from the preprint server.

