# Supplementary material for: From topography to connectome: Towards an integrated understanding of the resting brain
Source: bioRxiv. 2026 Jun 18:2026.06.06.730493. Originally published 2026 Jun 8. Preprint. [Version 2] doi: 10.64898/2026.06.06.730493 (PMC13277838; doi:10.64898/2026.06.06.730493)
Supplement: Supplementary file 1 [file NIHPP2026.06.06.730493v2-supplement-1.pdf]

bioRxiv preprint doi: https://doi.org/10.64898/2026.06.06.730493; this version posted June 18, 2026. The copyright holder for this preprint (which was not certified by peer review) is the author/funder, who has granted bioRxiv a license to display the preprint in perpetuity. It is made available under a CC-BY 4.0 International license.

Supplementary Materials

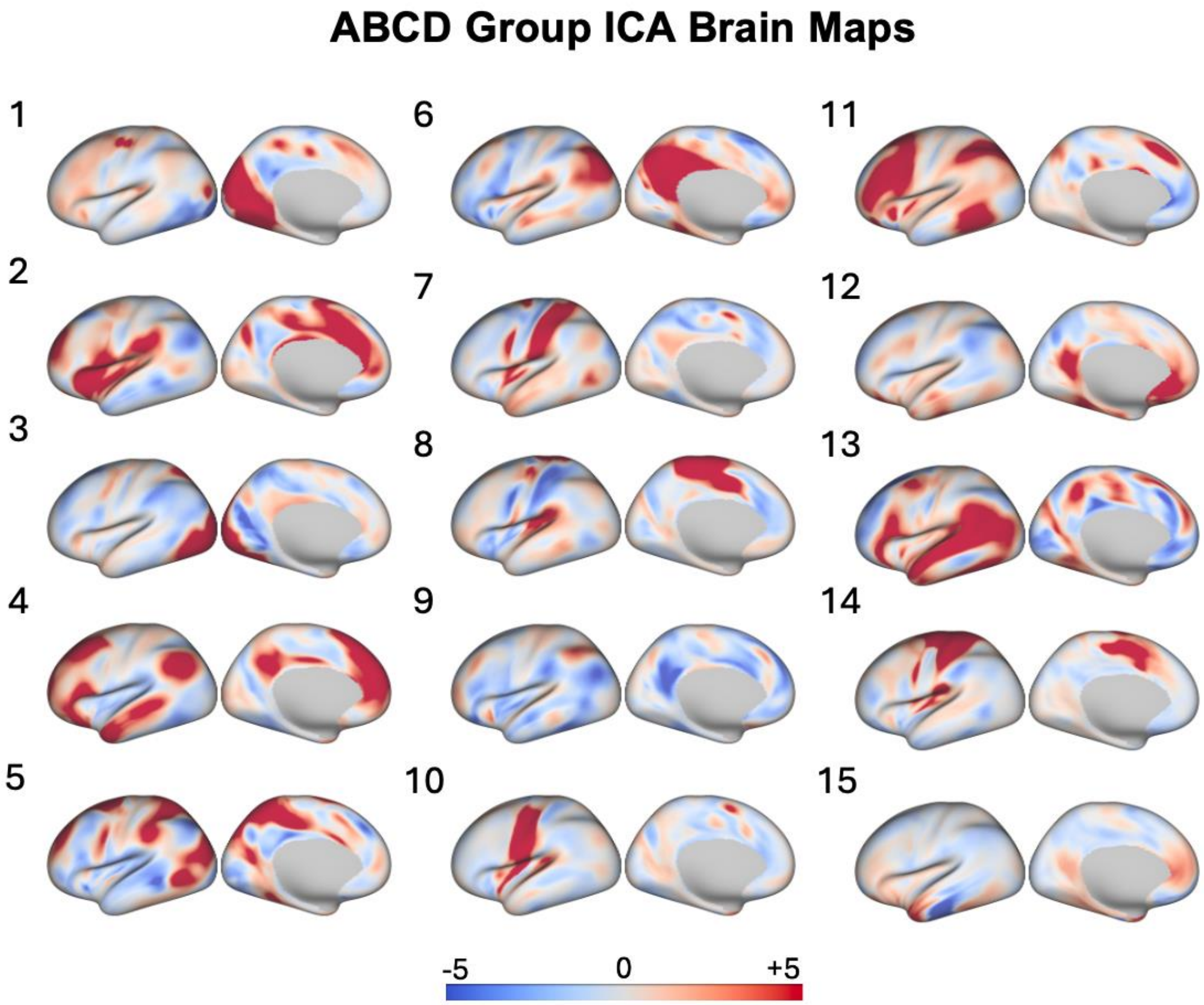

Figure S1. Group ICA maps for ABCD cohort. All ICA brain maps were derived using the FSL toolbox. Melodic was used to decompose rsfMRI scans into 15 independent components.

bioRxiv preprint doi: https://doi.org/10.64898/2026.06.06.730493; this version posted June 18, 2026. The copyright holder for this preprint (which was not certified by peer review) is the author/funder, who has granted bioRxiv a license to display the preprint in perpetuity. It is made available under a CC-BY 4.0 International license.

Supplementary Table 1. Topography-to-connectome model selection criteria across translations. To reduce overfitting to the training data, a separate held-out validation sample (N_{val} = 920) was used to compute MSE and MAE. Only models with the lowest MSE and MAE metrics were saved and used for testing. Performance of each selected model on the validation sample is shown on the table for all translations. All models were trained on 10 Intel Xeon Gold 6226R CPUs.

	**Schaefer-100**	**Schaefer-100 PC**	**Schaefer-300**	**Glasser-360**
**MSE**	0.025 ± 0.009	0.002 ± 0.001	0.028 ± 0.011	0.028 ± 0.011
**MAE**	0.123 ± 0.021	0.033 ± 0.009	0.129 ± 0.022	0.129 ± 0.022

bioRxiv preprint doi: https://doi.org/10.64898/2026.06.06.730493; this version posted June 18, 2026. The copyright holder for this preprint (which was not certified by peer review) is the author/funder, who has granted bioRxiv a license to display the preprint in perpetuity. It is made available under a CC-BY 4.0 International license.

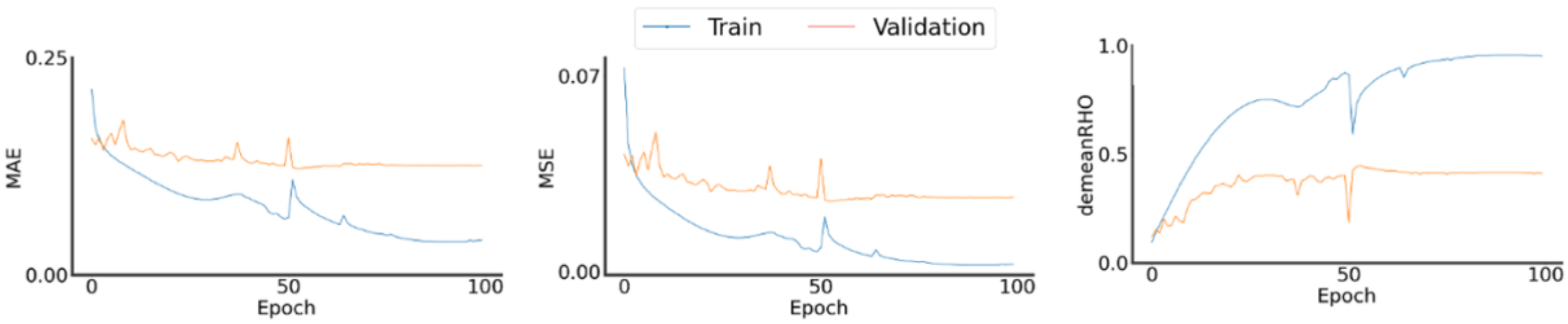

Figure S2. Model Training and Optimization Metrics. Mini-batch training epochs were used to train the topography-to-connectome translation model. MAE and MSE were used to assess performance on a held-out validation dataset. The best MSE model was chosen for all analyses and illustrations. Demeaned correlation results show steady increases as MAE and MSE decrease, but plateau at around the midpoint of training.

bioRxiv preprint doi: https://doi.org/10.64898/2026.06.06.730493; this version posted June 18, 2026. The copyright holder for this preprint (which was not certified by peer review) is the author/funder, who has granted bioRxiv a license to display the preprint in perpetuity. It is made available under a CC-BY 4.0 International license.

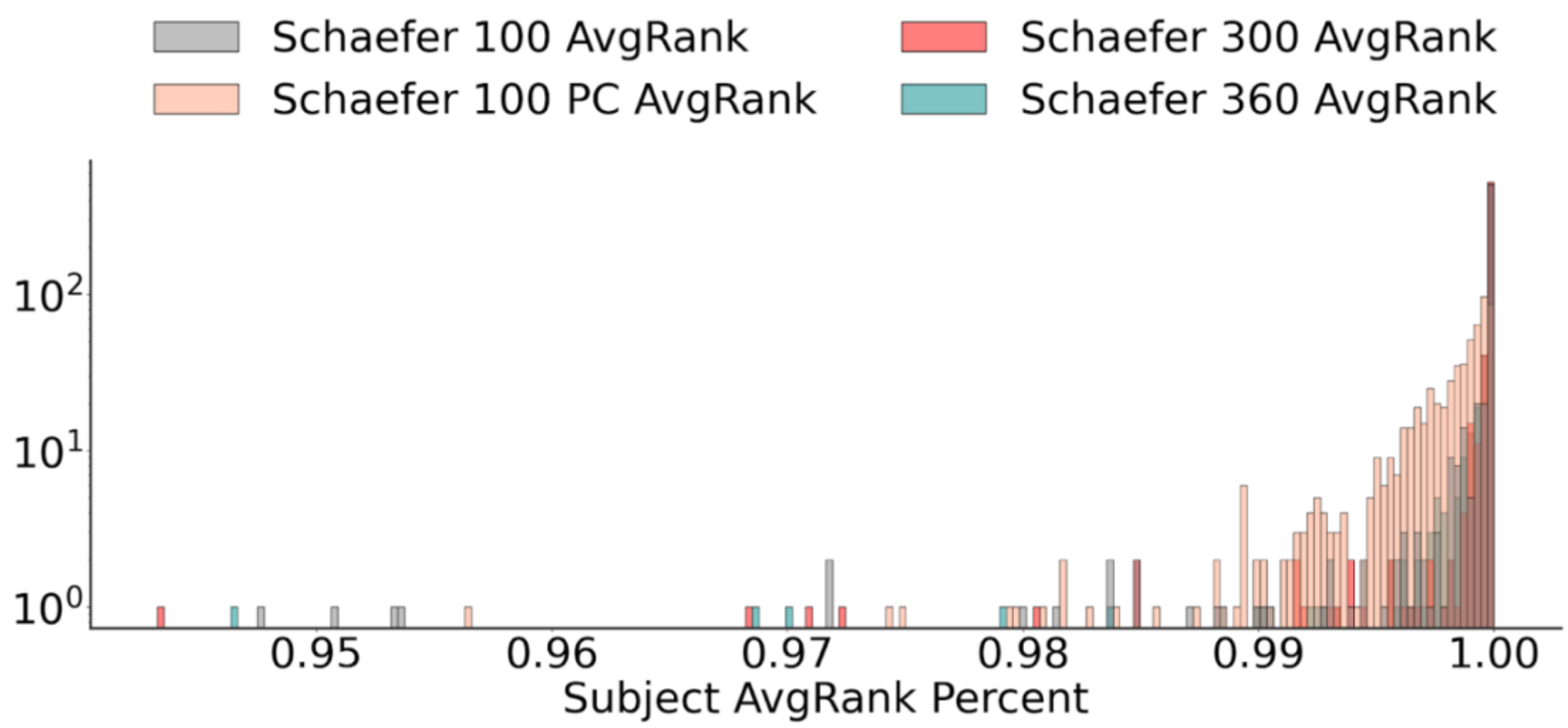

Figure S3. Average rank percentiles for all connectome types. Subject count on a logarithmic scale for average rank percentiles across all connectome types. A percentile of 100% (1.00) is equivalent to perfect subject finger printing based on translated connectomes. All percentiles are based on a held-out dataset of $N_{test} = 624$.

bioRxiv preprint doi: https://doi.org/10.64898/2026.06.06.730493; this version posted June 18, 2026. The copyright holder for this preprint (which was not certified by peer review) is the author/funder, who has granted bioRxiv a license to display the preprint in perpetuity. It is made available under a CC-BY 4.0 International license.

Supplementary Table 2. Topography-to-connectome model performance for Schaefer-100 translations across different ICO resolutions. All models were assessed with MSE and MAE during training. The table shows lowest MSE and MAE metrics achieved by the models during training on the validation sample (N_{val} = 920) across different ICO resolutions. ICO-04 inputs were too computationally demanding, such that a model could not be trained. All models were trained on 10 Intel Xeon Gold 6226R CPUs. OOM = Out Of Memory.

	**ICO-01**	**ICO-02**	**ICO-03**	**ICO-04**
**MSE**	0.025 ± 0.01	0.025 ± 0.01	0.04 ± 0.02	OOM
**MAE**	0.123 ± 0.02	0.123 ± 0.02	0.15 ± 0.03	OOM
**RHO (demeaned)**	0.43 ± 0.09	0.45 ± 0.09	0.32 ± 0.09	OOM
